# Using Paid and Free Facebook Methods to Recruit Australian Parents to an Online Survey: An Evaluation

**DOI:** 10.2196/11206

**Published:** 2019-03-06

**Authors:** Shannon K Bennetts, Stacey Hokke, Sharinne Crawford, Naomi J Hackworth, Liana S Leach, Cattram Nguyen, Jan M Nicholson, Amanda R Cooklin

**Affiliations:** 1 Judith Lumley Centre School of Nursing and Midwifery La Trobe University Bundoora Australia; 2 Murdoch Children's Research Institute Parkville Australia; 3 Parenting Research Centre Melbourne Australia; 4 Australian National University Canberra Australia

**Keywords:** parents, research subject recruitment, retention, social media, cost effectiveness, sampling bias, fathers, mothers, survey

## Abstract

**Background:**

The prevalence of social media makes it a potential alternative to traditional offline methods of recruiting and engaging participants in health research. Despite burgeoning use and interest, few studies have rigorously evaluated its effectiveness and feasibility in terms of recruitment rates and costs, sample representativeness, and retention.

**Objective:**

This study aimed to determine the feasibility of using Facebook to recruit employed Australian parents to an online survey about managing work and family demands, specifically to examine (1) recruitment rates and costs; (2) sample representativeness, compared with a population-based cohort of parents; and (3) retention, including demographic and health characteristics of parents who returned to complete a follow-up survey 6 weeks later.

**Methods:**

Recruitment was conducted using 20 paid Facebook advertising campaigns, supplemented with free advertising approaches such as posts on relevant Facebook pages and requests for professional networks to circulate the survey link via Facebook. Recruitment rates and costs were evaluated using the Checklist for Reporting Results of Internet E-Surveys, including view rate, participation rate, completion rate, cost per consent, and cost per completer. Sample representativeness was evaluated by comparing demographic and outcome variables with a comparable sample from the Longitudinal Study of Australian Children including educational attainment, marital status, country of birth, neighborhood disadvantage, work-family conflict, and psychological distress. Retention was evaluated by comparing the number and demographic characteristics of participants at recruitment and at 6-week follow-up.

**Results:**

Recruitment strategies together resulted in 6653 clicks on the survey link, from which 5378 parents consented to participate and 4665 (86.74%) completed the survey. Of those who completed the survey, 85.94% (4009/4665) agreed to be recontacted, with 57.79% (2317/4009) completing the follow-up survey (ie, 43.08% [2317/5378] of parents who consented to the initial survey). Paid Facebook advertising recruited nearly 75% of the sample at Aus $2.32 per completed survey (Aus $7969 spent, 3440 surveys completed). Compared with a population-based sample, participants at baseline were more likely to be university educated (*P*<.001), experience greater work-family conflict (*P*<.001) and psychological distress (*P*<.001), and were less likely to be born outside Australia (*P*<.001) or live in a disadvantaged neighborhood (*P*<.001).

**Conclusions:**

Facebook provided a feasible, rapid method to recruit a large national sample of parents for health research. However, some sample biases were observed and should be considered when recruiting participants via Facebook. Retention of participants at 6- to 8-week follow-up was less than half the initial sample; this may reflect limited ongoing participant engagement for those recruited through social media, compared with face-to-face.

## Introduction

### Overview

Busyness and time pressure are linked to reduced research participation [1], particularly for parents balancing the demands of work and family simultaneously [2]. This presents barriers to participation in health research, which is often time-intensive or requires face-to-face engagement. Social media platforms such as Facebook have the potential to reach parents who, as a cohort, are already strongly engaged in social media for parenting information, advice, or peer support [3,4]. Employed parents typically report high levels of time pressure [5]; therefore, online methodologies may allow more frequent and flexible opportunities for parents to engage in research. Although the omnipresence of social media in modern society offers new opportunities for efficient and low-cost participant recruitment, rigorous evaluation of Facebook as a recruitment method is scant and many unanswered questions remain. How feasible is this method for health research? Can it replace traditional offline methods of participant recruitment? Are samples of parents recruited via Facebook comparable to those recruited via offline methods? What are the retention rates for a convenience sample of parents recruited through Facebook? We address these questions salient to many researchers in a variety of health- and family-related fields and report on the methodology and feasibility of using Facebook to recruit employed Australian parents to an online survey, with a brief follow-up.

### Background

Facebook is a free social networking website whereby users create profiles, share content, and connect with other users. It remains the most popular social media platform globally, with over 2 billion active monthly users [6], including 15 million Australians, of whom 50% log in daily [4,7]. Facebook is the main social media platform used by parents [8], attracting more parents than nonparents on a daily basis [9]. In recent years, parents’ use of social media has increased [10], providing new ways for parents to maintain social ties, connect with other parents, share experiences, seek social and emotional support, and obtain parenting information [9,11]. Mothers typically use Facebook more frequently than fathers, and usage tends to increase during the transition to parenthood [3,9].

Given such widespread use of social media (and the ubiquity of smartphones), it is unsurprising that researchers are beginning to harness Facebook as a mobile, flexible, and potentially low-cost research tool. Traditional offline methods such as mail-outs and telephone interviews are becoming less feasible and less effective, evidenced by rising postage costs, increased refusal rates for household surveys, and reduced use of landline phones [12-14]. Extant literature varies enormously with respect to Facebook recruitment rates and costs. For example, Leach et al [15] recruited 819 new mothers to a health and well-being survey using Facebook advertisements in just 4 days, at a cost of Aus $0.55 per participant. Conversely, Kapp et al [16] used Facebook advertisements to recruit women to a survey of mammogram use but failed to recruit any eligible respondents, despite offering monetary incentives. Such variability likely reflects multiple factors such as the salience of the research topic, Facebook use among the target population, how the advertisement is presented, as well as the provision of incentives. A recent systematic review highlighted mixed results from Facebook recruitment studies [17]. From 54 studies, an average of 736 participants (range: 0 to 11799) were recruited at a cost of US $1.36 to US $110 per completing participant (per study average=US $17.48, of the 21 studies that provided sufficient data). Despite this variability, Facebook has been particularly effective for the recruitment of vulnerable and traditionally hard-to-reach populations, including young adults [18], HIV-positive participants [19], lesbians, bisexuals, gay, transgender, intersex, queer populations [20], new mothers [15], and low-income populations [21].

Although the use of Facebook for recruitment has grown dramatically in recent years, sample representativeness remains underreported. Thornton et al [17] found that only 16 (14.5%) studies examined sample representativeness, of which 86% concluded that their Facebook sample was similar to samples recruited using traditional methods. Leach et al [15] found that, compared with a representative population sample of mothers, mothers recruited via Facebook were younger and more likely to be in a de facto relationship, highly educated, first-time mothers, and speak only English at home. Importantly, these mothers also had poorer self-reported physical and mental health, suggesting potential bias in who self-selects into research participation about specific topics. Thornton et al [17] conclude that although the majority of Facebook samples have similar demographic characteristics to samples recruited via other methods, they are often not representative when compared with national population data. Evidence regarding the retention of samples recruited via Facebook is similarly limited. A 1-month follow-up of young adult veteran drinkers reported nearly 80% retention, with highly educated participants less likely to drop out [22].

### This Study

Rigorous evaluation of the feasibility of Facebook recruitment remains scant. In particular, there is a lack of explicit detail about Facebook advertising settings and strategies, content of advertisements, or modifications to Facebook settings and advertising campaigns throughout recruitment (exceptions include the studies by Leach et al and Arcia [15,23]). It is, therefore, difficult to draw accurate conclusions about the feasibility and effectiveness of Facebook as a research tool. To address these gaps, we conducted a methodological evaluation to assess the feasibility of using Facebook to recruit a national sample of employed parents to an online survey, with respect to (1) recruitment rates and costs, (2) representativeness (compared with a national population-based cohort of employed Australian parents), and (3) retention (including retention rate and demographic characteristics of those who returned to complete a follow-up survey 6 to 8 weeks later).

## Methods

The evaluation reported here is based on the protocol and data collected for an online survey, the Families at Work Survey. We first describe this survey and the recruitment protocol, followed by the evaluation method.

### The Families at Work Survey

The Families at Work online survey aimed to identify the employment conditions and workplace supports accessed by employed parents of children aged 0 to 18 years to manage work and family demands and to identify which strategies were associated with better parent well-being. Participants were required to be (1) aged 18 years or older, (2) living in Australia, (3) in paid employment (including self-employment or employees currently on leave), and (4) the parent of at least one child aged 18 years or younger. Upon survey completion, parents were invited to be contacted for a 6- to 8-week follow-up survey. The 15-min baseline survey (T1) was conducted between August and November 2016 (including school-term and holiday periods in Australia). The 10-min follow-up survey (T2) readministered core demographics and primary outcome measures and was conducted from October 2016 to February 2017.

Participants were invited to enter a draw to win 1 of the 10 Aus $50 gift cards at the end of each survey; winners were randomly selected and sent a gift card via email. The survey was administered via Qualtrics (Qualtrics Provo) [24]. Ethical approval was granted by La Trobe University Human Ethics Committee (S16-122).

### Recruitment Protocol

A study-specific Facebook page was created before recruitment that included a study description and contact details of the research team. The page featured the university logo to support the perceived legitimacy of the survey. Relevant content was regularly posted to the page, such as news articles about managing the demands of work and family life and updates on the number of survey respondents. Participants were recruited through either (1) paid Facebook advertising or (2) free Facebook advertising. Both methods included passive *snowball* sampling, as users liked, shared, or circulated the link to others. Survey advertisements comprised the following: (1) a title (eg, “When it comes to balancing work and family life, what works for you? What do you find tricky?”), (2) an image (eg, mother hugging child, father walking child to school), (3) main text (eg, “Researchers are looking for working parents of children aged 18 years or younger to complete a 15-minute survey. You can go into a draw to win a $50 gift card!”), and (4) the survey link.

### Paid Facebook Advertising

A total of 20 paid advertising campaigns were run at an overall cost of Aus $7969, approximately 1 campaign per week, most of which ran for 7 days each. A Facebook *campaign* has an overarching objective (eg, increase clicks to a website), targets specific Facebook users (eg, gender, age, location), and has a budget [25]. Data collection ceased after the 20th campaign due to a sufficiently large and diverse sample being obtained. The study was also advertised for a small fee (Aus $200) on a popular online single-parent community, whereby an administrator promoted the survey to members via email and across numerous single-parent Facebook pages.

Each paid advertising campaign was designed using Facebook’s Ads Manager, for which we selected the intended audience, schedule, format, and budget. Advertisements were displayed to users whose profiles indicated that they lived in Australia, were aged 18 to 60 years, and who matched on specific demographics, interests, or behaviors (eg, *mother*, *father*, *work-life balance*). Campaigns either targeted *parents* (all) or specifically targeted an underrepresented subgroup of our sample (eg, *fathers*, *fathers with teens*). For campaigns that specifically targeted mothers or fathers, users who identified their gender as *female* or *male* were selected in the Facebook Ads Manager to ensure that the advertisement was only shown to female or male users, respectively (Note: we have used the terms *gender*, *male*, and *female* for consistency with the Facebook Ads Manager, although we acknowledge the complexity and distinctions between sex, gender, and parenting roles). Campaigns targeting parents more broadly did not specify gender. A range of high-resolution stock images were used in the advertisements. Campaigns were closely monitored throughout data collection to identify subgroups of parents who were under-represented in the sample. No adjustments were made to campaigns once they had commenced. Campaigns were not run simultaneously, to avoid competition for the same target population (however, some overlap occurred with mother- and father-specific campaigns, given that these were targeting different populations). Features of the 20 campaigns are presented in Table 1. All advertisements were placed on Mobile News Feeds and Desktop News Feeds, given that this placement is highly visible and generates the strongest engagement at the lowest cost [26,27]. Users who viewed the paid advertisement on their News Feed could choose to *like*, *tag*, or *share* it with their friends through a passive *snowballing* approach.

**Table 1:** Characteristics of the 20 paid Facebook campaigns.

Number	Target	Image description	Format^a^	Duration (days)	Prize draw advertised	Time scheduling
1	Parents (all)	Including family, tradespeople, and florist at work	C	7	No	Anytime
2	Blue-collar workers	Including chefs, hairdresser, and factory worker	C	7	No	Anytime
3	Fathers	Including father doing laundry and tradesman at work	C	7	No	Anytime
4	Parents (all)	Including mother and son, father and daughter, and family	C	7	No	Anytime
5	Parents (all)	Mother and daughter and father and son	S	4	Yes	Anytime
6^b^	Parents (all)	Mother and daughter, and father and son	S	7	Yes	12-2 pm; 7-10 pm
7	Fathers	Father and son playing soccer	S	7	Yes	12-2 pm; 6-10 pm
8	Parents (all)	Father carrying daughter on shoulders	S	7	Yes	12-2 pm; 6-10 pm
9	Fathers	Father helping children build a bird feeder	S	7	Yes	12-2 pm; 6-10 pm
10	Parents (all)	Father helping son ride his bike to school	S	7	Yes	12-2 pm; 6-10 pm
11	Fathers	Father and children with a dog beside river	S	7	Yes	Excluding 12-2 pm; 6-10 pm
12	Fathers	Father and children with a dog beside river	S	7	Yes	Excluding 12-2 pm; 6-10 pm
13	Parents (all)	Mother and children	S	7	Yes	12-2 pm; 6-10 pm
14	Parents (all)	Father and son after a bike ride	S	7	Yes	12-2 pm; 6-10 pm
15	Fathers	Father and son playing football	S	5	Yes	Excluding 12-2 pm; 6-10 pm
16	Parents (all)	Mother helping son to do homework	S	7	Yes	12-2 pm; 6-10 pm
17	Fathers	Father and daughter reading a book	S	7	Yes	Excluding 12-2 pm; 6-10 pm
18	Mothers of teens	Mother and teenage son	S	7	Yes	12-2 pm; 6-10 pm
19	Fathers of teens	Father and teenage son	S	7	Yes	12-2 pm; 6-10 pm
20	Regional and rural parents	Family on cattle farm	S	7	Yes	Excluding 12-2 pm; 6-10 pm

^a^Carousel (C) format comprised a set of 5 scrolling images; single format (S) comprised 1 image.

^b^Campaign 6 was the first campaign using the revised survey landing page, with scheduled advertisements run at lunchtime (12-2 pm) and evenings (6-10 pm).

### Free Facebook Advertising

To supplement the paid advertising, we used *cross-* *promotion* to harness Facebook’s popularity and reach at no cost. We identified Facebook pages aimed at parents specifically and those used by the general adult population, by searching within Facebook or Google using keywords (eg, *parenting*, *mothers group*, *union*). We contacted page administrators to seek their support in promoting our survey link to their members or followers (see Multimedia Appendix 1 for example wording). The survey link was also circulated using an active *snowballing* approach, whereby the research team asked their professional networks to circulate the survey link through their personal Facebook account or within Facebook groups in which they were members.

### Survey Data Collection

Unique survey URLs were generated for each recruitment strategy and advertising campaign, which reflected the source of recruitment (eg, *Campaign 14*). This allowed us to monitor responses to each recruitment method. Facebook users who clicked on the survey link were directed to the survey landing page. For both T1 and T2 surveys, participants were asked to provide electronic consent by selecting 6 statements to demonstrate that they had read and understood the Participant Information Statement and agreed to participate in the survey. Participants who consented to be recontacted were emailed a unique survey link 6 to 8 weeks later. Nonresponders or participants who had partially completed the follow-up survey were sent up to 2 email reminders at weekly intervals.

### Evaluation Method

#### Facebook Metrics

Facebook metrics were collected through the Facebook Ads Manager, including reach (ie, the number of users who saw the adverts in their News Feed at least once), link clicks (ie, the number of users who clicked on the advertisement), cost per click (ie, campaign cost divided by the number of link clicks), and relevance (a score out of 10 generated by Facebook, which estimates how well the target audience is responding to the advertisement). Higher relevance scores indicate positive user engagement (eg, link clicks), whereas lower scores indicate negative interactions (eg, hiding or reporting an advertisement) [28].

#### Recruitment Rates and Costs

Recruitment rates and costs were calculated following the Checklist for Reporting Results of Internet E-Surveys (CHERRIES) [29]. The CHERRIES framework is designed to guide the quality reporting of online surveys in the same way as the Consolidated Standards of Reporting Trials statement guides the reporting of randomized controlled trials. As such, we report several metrics that speak to the quality and completeness of the data and of the overall feasibility of Facebook recruitment (that is, was a useable sample and dataset obtained?). These include the following: *view rate*: the ratio of Facebook users who clicked on the advertisement and visited the survey landing page divided by users who saw the advertisement (click to reach ratio); *participation rate*: the ratio of those who consented to participate divided by the number of visitors to the survey landing page (consent to click ratio); and *completion rate*: the ratio of the number of people who completed the survey divided by those who consented to participate (completion to consent ratio). Cost per consent and cost per completer for each campaign were also derived. It was not possible to calculate the view rates or response rates for our free recruitment strategies.

#### Determining Sample Representativeness

Comparison data were drawn from the Longitudinal Study of Australian Children (LSAC), Kindergarten (K) cohort (child age: 4-5 years at recruitment in 2004). Full sample details, design, and field methods are published elsewhere [30]. Briefly, LSAC employed a 2-stage cluster sampling design using Australian postcodes and Australia’s universal health insurance database (Medicare Australia) to recruit parents through a mailout. The LSAC sample is considered broadly representative of all Australian children and their parents. Data are collected biennially (since 2004) via a face-to-face interview with parents and a parent-report questionnaire [31,32]. Data from employed parents were compared with this study’s sample on baseline demographic characteristics and primary outcome measures at 3 waves: wave 1 (child age: 4-5 years), wave 4 (child age: 10-11 years), and wave 6 (child age: 14-15 years).

The demographic characteristics used for comparison were *marital status* (married or de facto; single); *country of birth* (born outside Australia; born in Australia); *education* (with or without tertiary qualification); and *neighborhood disadvantage*, assessed using the Socio-Economic Index of Areas (SEIFA) Disadvantage score [33]. Postcodes provided by the participants were matched with the corresponding SEIFA score (Australian *mean* 1000). To assess for self-selection bias, we also compared participants on the 2 main survey measures: work-family conflict and psychological distress. *Work-family conflict* was measured using 4 items on a 5-point scale, from 1=“strongly disagree” to 5=“strongly agree”, adapted from Marshall and Barnett [34] and used widely in Australian research (eg, [35,36]). Scores across the 4 items were averaged, with higher scores indicating greater work-family conflict (alpha=.67). *Psychological distress* was assessed using the K6 [37] on a 5-point scale, from 1=“none of the time” to 5=“all of the time”. Responses were summed (range 6-30), with higher scores indicating greater psychological distress (alpha=.87).

### Statistical Analyses

To determine Facebook recruitment rates and costs (Aim 1), survey data were exported from Qualtrics into StataSE14 (StataCorp) [38] and the number of consenting participants and completed surveys were summarized by the recruitment source (identified by the unique survey URLs). Facebook campaign costs were then summarized by the number of participants who provided consent (cost per consent) and the number of participants who completed the survey (ie, reached the end of the survey and clicked *submit*; cost per completer). To assess sample representativeness (Aim 2), baseline (T1) demographic characteristics were compared with LSAC waves 1, 4, and 6. Only employed parents from the LSAC sample were used, to provide a meaningful comparison. Independent sample *t*-tests were used to compare continuous variables (ie, work-family conflict, psychological distress, neighborhood disadvantage), and chi-square tests were used to compare categorical variables (ie, educational attainment, marital status, country of birth). To assess participant retention (Aim 3), the number of participants who completed the follow-up survey (ie, reached the end of the survey and clicked *submit*) were compared with those who consented to be recontacted and with those who consented to the initial study. Demographic characteristics of T1 and T2 participants were compared using independent samples *t*-tests and chi-square tests, as appropriate.

## Results

### Survey Recruitment (T1)

After a 15-week recruitment period, there were a total of 6653 clicks on the survey link, resulting in 5378 eligible participants consenting and commencing the T1 survey. Of these 5378, 4665 (86.74%) participants completed the survey (ie, pressed *submit* at the end of the survey) and a further 532 (9.89%) participants provided partial data (ie, exited before pressing *submit*); however, 181 (3.37%) participants provided consent but did not answer any questions. The proportion of consenting participants who provided complete, partial, or no data did not differ for the paid and free methods. Of the 4665 participants who provided complete data, 3440 (73.74%) were recruited through the 20 paid Facebook advertising campaigns, 79 (1.69%) through other paid online advertising, 782 (16.76%) through free Facebook advertising, and 364 (7.80%) through circulation within the Facebook accounts of our professional networks.

**Figure 1:**
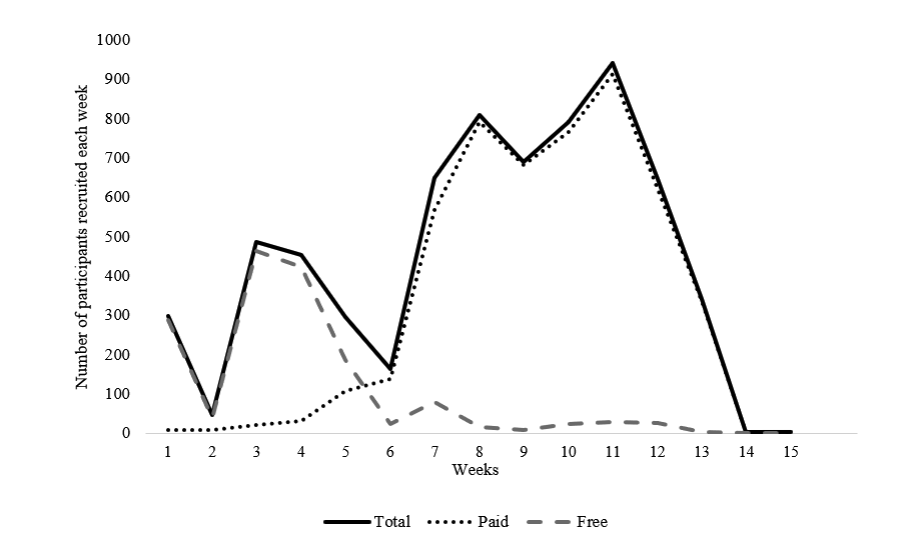
Paid versus free recruitment rates during the T1 survey.

**Figure 2:**
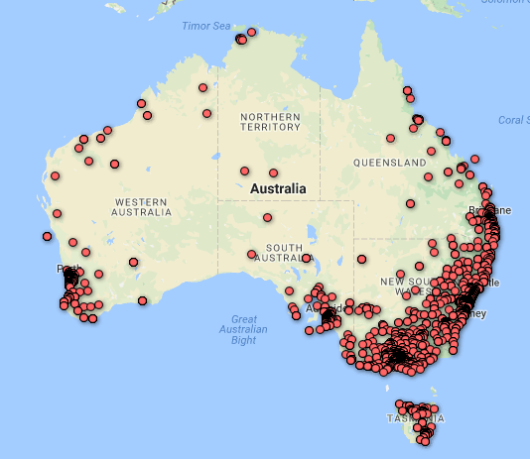
Geographical distribution of participants (for 5213 participants who provided postcodes).

As shown in Figure 1, free advertising was the main source of recruitment during the first 5 weeks of data collection. After this time, our paid advertising campaigns had been fine-tuned to increase effectiveness and thus became the main recruitment method. This allowed a reduced focus on free methods, given that we had exhausted opportunities to post on pages with which we had established connections. In weeks 3 and 7, efforts to encourage circulation of the survey link within personal Facebook accounts of our professional networks were also effective. A pivotal point in recruitment can be observed around weeks 5 and 6, whereby changes to paid advertising strategies (described below) were implemented. Most participants completed the survey on a mobile device (72.80% [3396/4665]), followed by tablet (12.11% [565/4665]), laptop (8.00% [373/4665]), and desktop computer (7.01% [327/4665]). Respondents represented all Australian states and territories, with greater concentrations in or around the more populous cities of Melbourne, Sydney, Brisbane, Adelaide, Perth, and Hobart (see Figure 2).

### Paid Facebook Advertising

The 20 paid Facebook advertising campaigns reached nearly half a million users, with a view rate (click to reach ratio) of 1.87%, a participation rate (consent to click ratio) of 47.80%, and a completion rate (completion to consent ratio) of 86.04%. The total cost of the paid Facebook advertisements was Aus $7969.25, with an average cost of Aus $1.99 per consenting participant and Aus $2.32 per completed survey. Table 2 summarizes the results of the 20 paid advertising campaigns, including the overall cost, reach, relevance, clicks, cost per click, cost per consenting participant, and cost per completed survey. As per our methodology, campaigns were regularly monitored, and subsequent campaigns were adjusted accordingly. The cost per consent started at Aus $31.79 and dropped to Aus $7.52 by Campaign 5. This decrease marked a shift in our recruitment strategies; we mentioned our incentive (ie, gift card prize draw) and changed from a multiple- to a single-image format. A further decrease in cost per consent occurred at Campaign 6 (Aus $2.54), after we reduced the text on the survey landing page and scheduled our advertisements to appear during specific time slots. Although cost per consent fluctuated across subsequent campaigns, it remained relatively low, ranging from Aus $0.68 (Campaign 14) to Aus $4.86 (Campaign 7) per consenting participant. Adjustments made to campaigns throughout data collection did not markedly change the cost per click but did improve the completion rates.

**Table 2:** Recruitment rates and costs for paid Facebook advertising campaigns.

Number	Cost (Aus $)	Reach^a^	Relevance score^b^	Link clicks (view rate), n (%)	Cost per click (Aus $)	Consented to participate (participation rate), n (%)	Cost per consent (Aus $)	Completed survey (completion rate), n (%)	Cost per completer (Aus $)
1	349.67	14,832	6	428 (2.89)	0.82	11 (2.57)	31.79	9 (81.82)	38.85
2	339.01	10,448	8	459 (4.39)	0.74	7 (1.53)	48.43	4 (57.14)	84.75
3	336.32	16,058	6	371 (2.31)	0.91	8 (2.16)	42.04	7 (87.50)	48.05
4	336.64	17,599	6	383 (2.18)	0.88	8 (2.09)	42.08	6 (75.00)	56.11
5	188.11	10,488	4	134 (1.28)	1.40	25 (18.66)	7.52	24 (96.00)	7.84
6	350.00	17,408	6	271 (1.56)	1.29	138 (50.92)	2.54	122 (88.41)	2.87
7	350.00	19,995	4	184 (0.92)	1.90	72 (38.59)	4.86	62 (86.11)	5.65
8	350.00	26,152	8	605 (2.31)	0.58	415 (59.11)	0.84	353 (85.06)	0.99
9	700.00	35,087	2	348 (0.91)	2.01	187 (51.72)	3.74	162 (86.63)	4.32
10	350.00	21,648	6	347 (1.60)	1.01	202 (58.50)	1.73	180 (89.11)	1.94
11	700.00	45,272	7	976 (2.16)	0.72	496 (50.82)	1.41	421 (84.88)	1.66
12	700.00	38,121	7	707 (1.85)	0.99	331 (46.68)	2.11	265 (80.06)	2.64
13	350.00	24,249	8	499 (2.06)	0.70	343 (68.94)	1.02	298 (86.88)	1.17
14	350.00	26,080	9	702 (2.69)	0.50	514 (73.65)	0.68	460 (89.49)	0.76
15	399.06	18,576	6	328 (1.77)	1.22	187 (57.62)	2.13	157 (83.96)	2.54
16	316.37	18,768	8	375 (2.00)	0.84	321 (62.93)	0.99	285 (88.79)	1.11
17	604.40	30,750	4	375 (1.22)	1.61	270 (48.27)	2.24	235 (87.04)	2.57
18	300.00	19,920	8	460 (2.31)	0.65	290 (64.57)	1.03	247 (85.17)	1.21
19	300.00	16,788	4	166 (1.00)	1.81	66 (42.17)	4.55	55 (83.33)	5.45
20	299.67	18,548	6	246 (1.33)	1.22	107 (43.90)	2.80	88 (82.24)	3.41
Total	7969.25	44,6787	—^c^	8364 (1.87)	—	3998 (47.80)	—	3440 (86.04)	—
Mean	—	—	6.2	—	1.50	—	1.9	—	2.32

^a^Reach is the number of users who saw the adverts in their News Feed at least once.

^b^Relevance score is out of 10 and estimates how well the target audience is responding to the advertisement; higher scores indicate positive engagement.

^c^Not applicable.

**Figure 3:**
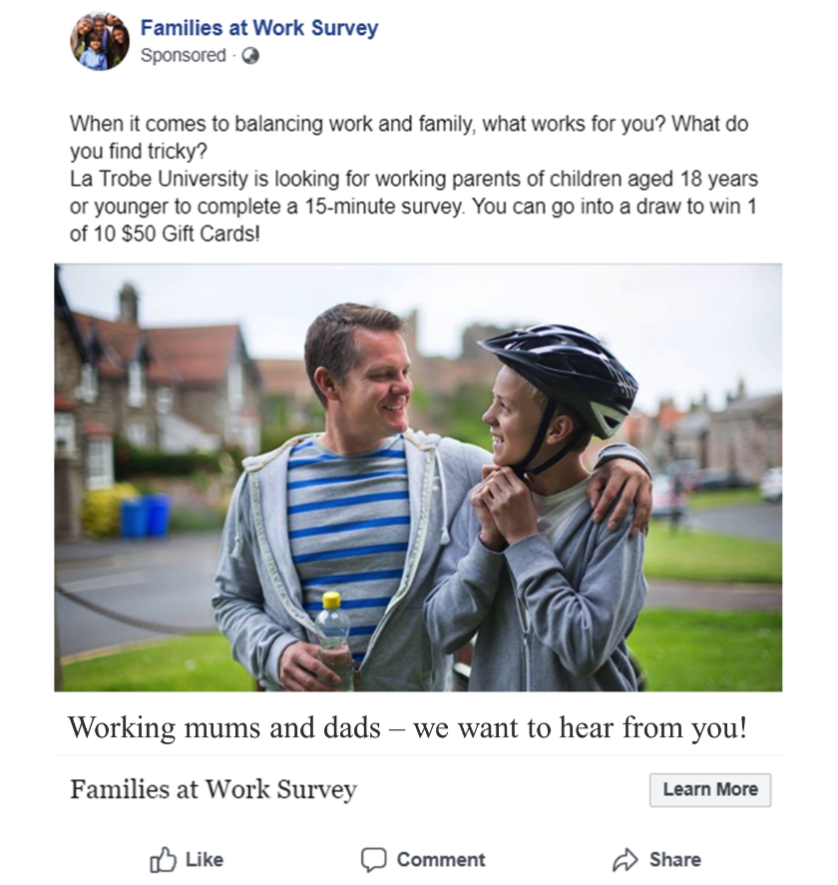
Most successful Facebook campaign (Campaign 14), which generated 514 survey consents at Aus $0.68 each (Aus $0.76 per completed survey) and a relevance score of 9 out of 10.

Although the changes implemented at Campaigns 5 and 6 improved the participation rates, we continued to monitor participant demographics and refine our advertising campaigns. For example, Campaign 6 targeted parents generally (ie, no gender specified), reaching 17408 users and attracting 271 link clicks. However, only 13.56% (2361/17,408) of views and 4.06% (11/271) of clicks were from male users. Overall, 8 of the 20 campaigns targeted male users (fathers), each of which recruited between 83.94% and 100% males. In comparison, the 11 campaigns in which no gender was specified, each recruited between 0% and 12.53% males. Thus, the father-specific campaigns effectively boosted participation from fathers; overall, the paid advertising campaigns recruited 1540 fathers (44.77% of the total sample recruited through the paid campaigns). Parents in regional Australia and parents of teenage children were also under-represented, prompting 3 new campaigns targeting regional parents, mothers of teens, and fathers of teens. The most successful paid campaign was Campaign 14 (Figure 3), which recruited 514 participants at a cost of Aus $0.68 per consented participant (ie, Aus $0.76 each for 460 completed surveys). This campaign coincided with *back to school* week, in which Australian children return to school after the holidays; the image was selected to maximize salience for parents during this period.

### Free Facebook Advertising

We contacted the administrators of 85 Facebook pages, from which 22 responded, 13 agreed to endorse and cross-promote the study on their Facebook page, 6 invited us to post directly on their page as a visitor, and 3 declined as they did not promote surveys on their page. We did not receive a response to 73% of requests. Of the 6 Facebook pages that provided permission to post as a visitor, 1 post led to the completion of 6 surveys, whereas 5 posts generated none. In comparison, 8 Facebook administrators endorsed our survey on their page, resulting in the completion of 492 surveys. The success of each endorsed post generally reflected its number of *followers*. For example, a prominent parenting Facebook page with almost 200,000 followers led to the completion of 355 surveys, whereas a parenting page with 3000 followers generated 11 surveys. In all, 4 administrators shared our survey link with their members via email (generating 70 surveys) and 1 posted the survey link in their forum (generating 138 surveys). An additional 76 parents completed the survey via the link displayed on our study-specific Facebook page. Our requests for professional networks to circulate the survey link within their own Facebook account and groups were effective, recruiting 364 participants with complete data (7.8% of the full sample).

### Retention (T2)

Of the 4665 participants who completed the T1 survey (ie, reached the end of the survey and clicked *submit*) 85.94% (4009/4665) agreed to be contacted for the follow-up survey. Of these, 2463 (61.44%) participants consented to the T2 survey: 35.74% (1433/4009) responded to the initial email invitation, 15.71% (630/4009) responded to the first email reminder, and 9.98% (400/4009) to the second. One-third (32.98%, 1322/4009) of the participants did not respond to the email invitation or either reminder, a further 4.81% (193/4009) did not provide an email address in the T1 survey despite agreeing to be recontacted, and 31 (0.77%) provided an invalid email address. A small number of participants who consented at T2 provided no data (0.65%, 16/2463) or partial data (5.28%, 130/2463). Of the 4009 participants who agreed to be contacted for follow-up, 57.79% (2317) completed the T2 survey (ie, 43.08% [2317/5378] of parents who consented to the initial survey).

Sample characteristics of participants who completed the T1 and T2 surveys were compared. There were similar proportions of single parents (14% T1 vs 15% T2; *P*=.55) and parents born outside Australia (18% T1 vs 19% T2; *P*=.57) across both surveys. Parent age was also similar (mean 40.3 T1 vs mean 40.4 T2; *P*=.53), as was neighborhood disadvantage (SEIFA score mean 1019 T1 vs mean 1020 T2; *P*=.26). More males completed the T1 survey, compared with the T2 survey (32% T1 vs 27% T2; *P*<.001). Finally, T1 participants experienced greater work-family conflict (mean 3.4 T1 vs mean 3.3 T2; *P*<.001) and greater psychological distress (mean 11.5 T1 vs mean 11.2 T2; *P*=.005) than T2 participants.

### Sample Representativeness

Demographic characteristics for T1 were compared with a representative sample of employed Australian parents participating in the LSAC. To compare across child age ranges, we repeated the comparisons with LSAC waves 1, 4, and 6. Similar findings were observed across each wave; therefore, only wave 1 (data collected in 2004) is reported here for comparative purposes (full results available on request). Compared with LSAC, the current sample consisted of more single parents (15% vs 14%; *P*<.001) and parents with tertiary education (65% vs 44%; *P*<.001) and fewer parents born outside Australia (18% vs 23%; *P*<.001). Parents in our sample also lived in less disadvantaged neighborhoods (SEIFA score mean 1018.7 vs mean 1011.1; *P*<.001) and reported greater psychological distress (mean 11.5 vs mean 10.1; *P*<.001) and greater work-family conflict (mean 3.4 vs mean 2.6; *P*<.001).

## Discussion

### Principal Findings

This paper reports on the feasibility of using Facebook to recruit a sample of working Australian parents to an online survey; this is one of few studies to systematically evaluate participant recruitment using social media for health research purposes in Australia (see also [15,27]). We report on the key parameters of recruitment rates and costs, sample representativeness, and retention, following the CHERRIES framework [29] for reporting online surveys. Our findings have implications for other researchers seeking to recruit participants through social media and also contribute to the emerging evidence about the nature of samples recruited using these methods.

Overall, the combination of paid and free Facebook advertising proved to be an effective strategy for recruiting a large sample of employed parents (4665 parents completed the survey over a 15-week period). After initially poor engagement with paid advertisements and high per-participant costs, adjustments to our paid advertisements resulted in significantly improved recruitment and participation rates (eg, advertising a prize draw incentive, reducing the amount of text on the survey landing page, using a single-image advertisement). These improvements reduced the need to focus our efforts on free methods. It should be noted that a relatively large proportion of participants (13.3% overall) indicated their consent but provided either no data or partial data. It has been reported elsewhere that participants recruited via social media may be less “conscientious” than those recruited using more traditional methods [18]. As such, we suggest that iterative methods and careful monitoring are required during Facebook recruitment.

We provide further support for the use of targeted Facebook recruitment as a low-cost means of recruiting parents [15,27,39]. Facebook may be a particularly versatile tool in today’s tight monetary environment, in which researchers are under increasing pressure to obtain competitive funding and minimize research costs [40]. Our average cost per survey was substantially lower than most of those conducted to date (paid campaigns only: Aus $2.32 per survey; overall: Aus $1.75 per survey), with the exception of Leach et al [15], who achieved Aus $0.55 for a survey targeting women postpartum. The success of the Leach et al survey may reflect the salience of the research topic (ie, the “Living with a Young Baby” survey) and the high rate of Facebook use among new mothers [3]. Our recruitment costs also compare favorably with more traditional recruitment methods. For example, the authors of this study previously recruited 2002 Australian parents to complete a survey via Computer Assisted Telephone Interview at approximately Aus $119 per participant (direct research costs only) [41].

Engagement with our paid Facebook advertising campaigns compares favorably with previous studies. Nearly 2% of Facebook users exposed to our advertisements clicked on the link, and of those who did, nearly half (47.80%) consented to participate. The average click-through rate (1.87%) was also higher than reported elsewhere [21,23,42]. However, as illustrated in Table 2, the number of Facebook users an advertisement reaches or the number of *likes*, *shares*, and *clicks* it generates does not necessarily translate to survey completion.

Our sample of working Australian parents was broadly representative, compared with a large population-based sample. Consistent with Leach et al [15], some self-selection bias was evident in that we recruited parents experiencing more of the constructs being examined (ie, work-family conflict, psychological distress). It is likely that parents who viewed our advertisement about “juggling work and family life” were more likely to respond if they perceived this to be a salient issue. Some demographic characteristics, although statistically significant due to the large sample size, were not meaningfully different (ie, proportions of single parents, neighborhood disadvantage). Our sample, however, did under-represent parents with lower educational attainment and parents born outside Australia, which is consistent with findings reported elsewhere [15,43]. Although 97% of Australian families with children under 15 years have internet access [44], those on low incomes are less likely to be internet users than those on high incomes, and migrants from non-English–speaking backgrounds are less likely to be online than their Australian-born counterparts [45]. It is possible that our method of harnessing professional networks to circulate the survey link over-recruited tertiary-educated parents; however, this tends to occur for both online and offline research methodologies [46].

Less than half of those initially recruited returned to complete the follow-up survey 6 to 8 weeks later, which is lower than the retention rates reported elsewhere (eg, [22]). This may reflect limited ongoing participant engagement for those recruited through social media, compared with face-to-face. Indeed, Frandsen et al [18] concluded that participants recruited via social media tended to be less invested or conscientious than those recruited via more traditional means such as flyers or newspapers. It is possible that some Facebook users impulsively click on a link out of curiosity, compared with more active engagement that might be required with traditional methods (eg, emailing the research team).

Facebook allows for a flexible and dynamic approach to recruitment, whereby strategies can be continually monitored, adjusted, and evaluated for effectiveness. This requires researchers to be highly responsive and open to trying different advertising strategies [46]. Although social media recruitment can lead to less contained or trackable recruitment than offline methods, users’ ability to *like*, *share*, or *tag* other users in response to an advertisement can support ongoing *snowballing* and broad reach. A particular strength of our evaluation method was the use of unique survey URLs for each advertising campaign [24]. This enabled us to identify the specific campaign a participant engaged with. Facebook also provides a multitude of metrics through its Ads Manager for users to track the effectiveness of advertising campaigns and engagement with posted content. We used a flexible approach to develop targeted campaigns to reach specific subgroups, such as parents of teenagers, blue-collar workers, and those living in rural or remote areas.

Fathers were more difficult to recruit than mothers, requiring specific, targeted campaigns with explicit calls to action. This may reflect fewer fathers engaging in Facebook or around parenting-related topics [3], but also reflects evidence that fathers are typically under-represented in research [47,48]. Interestingly, advertisements targeted at *parents* engaged few fathers, and it became apparent that fathers required *specific* calls to action if they were to take part. This may be due, in part, to fathers engaging with less parent-related Facebook content than mothers or that the term *parents* was viewed as a reference to mothers rather than fathers. It may also be a product of Facebook functionality, whereby the campaign is presented to users who are similar to those who have already engaged with it (ie, mothers). This necessitates the use of different recruitment strategies for mothers and fathers.

The gradual adjustment of advertising strategies across the duration of recruitment allowed us to identify the most effective means of engaging our target population. A single-image advertisement was more successful than the multiple-image *carousel* format, although Facebook reports that “carousel link ads drive 30-50% lower cost per conversion and 20-30% lower cost per click than single-image link ads” [49]. It is possible that a single relevant image was most salient to our population of working parents or that the *carousel* format is more effective for campaigns that aim to promote a range of goods or products.

### Limitations

We acknowledge several limitations of our evaluation of Facebook for recruitment. First, we made several changes to our recruitment strategy for campaigns 5 and 6, which resulted in a substantial improvement in participant recruitment rates and per-participant costs. We acknowledge that this change in format coincided with the decision to specifically mention our prize draw in the advertisement. It is, therefore, difficult to disentangle the relative contributions of these strategies to recruitment rates. Second, we compared our sample with a large cohort study (LSAC), and although it is considered broadly representative of the Australian population, it is not a contemporary sample given that wave 1 data were collected in 2004. Third, evaluation of Facebook as a recruitment tool can be impacted by its advertising algorithms and metrics, which are often difficult to comprehend and can change without notice [50]. This can pose problems for researchers interpreting metrics or seeking to replicate previously published Facebook recruitment protocols. In January 2018, Facebook founder, Mark Zuckerberg, announced a new algorithm that would result in “less public content like posts from businesses, brands and media” [51]; such changes are likely to impact the way in which Facebook is used for research. The constant evolution of Facebook also makes between-study comparisons problematic, which is exacerbated by the lack of consistency with which existing studies report on key parameters such as cost (eg, studies may report total cost, cost per click, cost per participant, or cost per completer).

### Practical Challenges

We encountered several practical challenges during recruitment. Both paid and free methods were surprisingly time-intensive, requiring regular monitoring of response rates (daily in the first few weeks, reducing to weekly by the end of the recruitment period), designing new campaigns, contacting page administrators, and developing content for the study Facebook page. Currently, Facebook allows page administrators to block posts but not comments. Our decision to set the profanity filter to *strong* and to use a large selection of *moderation words* was effective; only a small number of offensive or negative comments were posted and were automatically hidden, keeping administrative requirements to a minimum. During the early phase of recruitment, our private messages to Facebook page administrators about promoting our survey were labeled as *spam*. Our account was subsequently blocked from posting or messaging for 1 month; this required the creation of a new account, to enable active recruitment to continue. Facebook does not provide any direct support service; therefore, we needed to rely on Facebook forums or the expertise of peers. Another challenge was the low response rate from administrators of Facebook pages regarding requests to support our research by posting the survey link. It is possible that some Facebook pages or groups receive many requests from researchers or that our message was simply disregarded as *spam*. A preexisting connection with the group or page generally led to a greater likelihood of a response; therefore, researchers are encouraged to draw on personal or professional networks and to contact individuals directly (eg, via email) where possible. We also recognize that this free advertising is not necessarily a sustainable approach in the long term, given the potential risks of survey fatigue or of oversampling specific subgroups of the population.

### Further Research and Recommendations

Future evaluations of Facebook recruitment may seek to harness qualitative methodologies to understand the reasons participants choose to engage or not engage in research advertised on Facebook, including the features of advertising campaigns and survey interfaces that may be most appealing for specific target groups, and the factors that may improve participant retention. This would be particularly helpful for fathers, who were more difficult than mothers to recruit and retain. It is also recommended that recruitment strategies are amended sequentially, rather than simultaneously, to identify the effectiveness of specific strategies with greater precision. Although we found Facebook to be an effective tool for the recruitment of working Australian parents, further research is needed to determine its feasibility for nonparent or unemployed populations and to examine the factors affecting retention following Facebook recruitment.

We recommend that careful consideration is given to engaging participants at each step of the way from viewing the advertisement, providing consent, to survey completion. An advertisement must be relevant and interesting; the survey landing page must be clear and concise with friendly, plain language; and the survey itself must be straightforward and not unreasonably lengthy. As documented elsewhere [46], regular communication with an institutional human research ethics committee is essential, from the study design phase and throughout the recruitment phase. However, the online space is remarkably dynamic, with social media platforms and functionality often changing rapidly and unpredictably. Researchers must stay on the cutting edge of what platforms are popular and how they function. Seeking guidance from an information technology or social media specialist during project design and implementation may be beneficial.

### Conclusions

Findings suggest that Facebook has the potential to be a low-cost means of recruiting a large sample of working Australian parents, which is an important consideration given the competitive funding environment in which researchers work. A significant barrier is the ever-changing nature and functionality of social media; researchers may benefit from the support of social media professionals. Although we focus on the recruitment of parents, our methodology is applicable to the recruitment of other populations, providing access to real-time feedback and allowing recruitment gaps to be addressed using targeted campaigns. Our experience suggests that immediate success is unlikely; rather, sufficient lead-in time is required to build interest and momentum and to monitor and adjust recruitment strategies accordingly. Fathers were unlikely to respond to calls for *parents* but required specific invitations to *dads* using gender-specific campaigns. There was evidence of self-selection bias, given that we recruited parents with greater work-family conflict and psychological distress than the general parent population. Participants were also more highly educated and less likely to have been born outside of Australia than the general parent population. Retention to follow-up was less likely for males or for participants experiencing high work-family conflict and psychological distress. Further evidence is needed to understand the mechanisms of engagement and retention for populations recruited through social media.

## Appendix

Multimedia Appendix 1 Example messages sent to Facebook page administrators.

## Abbreviations

CHERRIES: Checklist for Reporting Results of Internet E-Surveys
LSAC: Longitudinal Study of Australian Children
SEIFA: Socio-Economic Index of Areas
T1: Time point 1 (ie, baseline survey)
T2: Time point 2 (ie, follow-up survey)
