# Supplementary material for: Using Paid and Free Facebook Methods to Recruit Australian Parents to an Online Survey: An Evaluation
Source: J Med Internet Res. 2019 Mar 6;21(3):e11206. doi: 10.2196/11206 (PMC6425313; doi:10.2196/11206)
Supplement: Multimedia Appendix 1 [file jmir_v21i3e11206_app1.pdf]

Appendix 1: Example Messages Sent to Facebook Page Administrators

Longer Version

Hello,

Our team here at La Trobe University has just launched an online survey called “Families at Work”. The survey aims to understand the support needs of Australian parents to manage work and family demands. It’s a brief 15-minute online survey and we imagine this would be a salient and engaging topic for many of your “followers”.

We are wondering if you may consider supporting us by posting a link to our survey on your Facebook Page? If you would like any further information about our survey, we also have a Facebook Page, which can be accessed at: [link here].

This project has ethical approval from La Trobe University (SHE-CHESC reference number: S16-112).

If you able to support our study, here is a brief blurb and survey link you may wish to post on your page:
“La Trobe University researchers are looking for Australian mothers and fathers who are currently employed in a paid job to take part in a 15-minute survey about juggling your work and family responsibilities.
Please click the link below to find out more: [link here]”.

Please don’t hesitate to let us know if you need any further information.

Kind regards,
Families at Work Team

Shorter Version

Hi [name here], we are a research group from La Trobe University in Melbourne looking at how parents manage work and family life. We would love to hear from dads about how they juggle work and family responsibilities.

It's just a brief 15-min survey - would it be possible for you to share this with your group? Please check out our "Families at Work" Page for further info.

The survey can be accessed here: [link here]

Looking forward to hearing from you,
Families at Work Team
